# Assembly of inorganic [Mo_2_S_2_O_2_]^2+^ panels connected by selenite anions to nanoscale chalcogenide–polyoxometalate clusters†
†Electronic supplementary information (ESI) available. See DOI: 10.1039/c5sc04944j


**DOI:** 10.1039/c5sc04944j

**Published:** 2016-02-25

**Authors:** Hong-Ying Zang, Jia-Jia Chen, De-Liang Long, Leroy Cronin, Haralampos N. Miras

**Affiliations:** a WestCHEM , School of Chemistry , University of Glasgow , University Avenue , Glasgow , G12 8QQ , UK . Email: Charalampos.moiras@glasgow.ac.uk ; Email: lee.cronin@glasgow.ac.uk

## Abstract

We describe how supramolecular assembly, mediated by control of the ratio of the hetero-atoms in the units [Mo_2_S_2_O_2_]^2+^ and SeO_3_^2–^ leads to the formation of new types of building blocks, [(Mo_2_O_2_S_2_)_3_(OH)_4_(H_2_O)_6_(SeO_3_)] = {Mo_6_} and [(Mo_2_O_2_S_2_)_2_(OH)_2_(H_2_O)_4_(SeO_3_)] = {Mo_4_} which are linked in an type of inorganic ‘panelling’ to the assembly of a range of new clusters **1–3** with the general formula {(Mo_2_O_2_S_2_)_*x*_(OH)_*y*_(SeO_3_)_*z*_(H_2_O)_*w*_}^*n*–^, where *x*, *y*, *z*, *w*, *n* = [8, 0, 20, 8, 24] for **1**, [14, 14, 17, 8, 20] for **2** and [8, 8, 8, 0, 8] for **3**. Cluster **1**, a rare example of inorganic cryptand, exhibits an elliptical “endo” motif defining an anisotropic ellipse with the dimensions 1.7 × 1.0 nm, with pores ranging from 5.3 to 6.4 Å and site selective cation recognition properties; cluster **2** exhibits an “exo” structural motif constructed by 3 × {Mo_6_} and 2 × {Mo_4_} panels spanning a cross shape 2.4 × 2.0 nm and cluster **3** a ring shaped structure of a 1.5 nm in diameter. The control of endo *vs.* exo topology as a function of the Se : Mo ratio is reflected to the difference in surface area of *ca.* 500 Å^2^ between clusters **1** and **2** intermolecular interactions and proton conduction properties, and this work shows that very simple synthetic parameters can critically change the structure and properties of all-inorganic nanoscale chalcogenide–polyoxometalates.

## Abstract 2

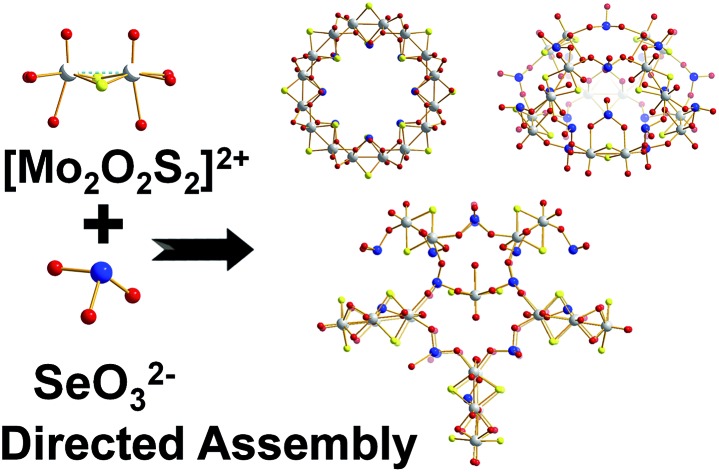
Supramolecular assembly of [Mo_2_S_2_O_2_]^2+^/SeO_3_^2–^ based building blocks, mediated by controlling the hetero-atom ratio, leads to the formation of clusters {(Mo_2_O_2_S_2_)_*x*_(OH)_*y*_(SeO_3_)_*z*_(H_2_O)_*w*_}^*n*–^ exhibiting ring, “exo” and “endo” architectures with multiple binding sites and cation recognition functionality.

## Introduction

Polyoxometalates (POMs) and polyoxo(thio)metalates have been the subject of a large number of studies due to their nanoscale size, versatile architectures and tuneable electronic and physical properties,^1^–^4^ as well as fundamental investigations of their supramolecular self-assembly.^5^,^6^ The observed structural diversity of POMs is driven by a wide range of combinatorially accessible structures, which constitutes a challenging problem when it comes to the reliable generation of new architectures in high yield.^6^,^7^ A representative example is the Molybdenum Blue (MB) family which includes the wheel-shaped {Mo_154_},7*a* {Mo_176_}8*a* and “lemon-shaped” {Mo_368_}8*b* species whose vast size has helped define a new class of gigantic inorganic architectures9*a* and inner pores which have allowed supramolecular host–guest reactions in an all inorganic host.9*b* In general terms, recent advances in the field of supramolecular chemistry have led to demonstrations of control over their assembly process, by utilizing organic structure directing ligands^10^ for the targeted synthesis of large structures.^11^ The promise is that understanding of the synthetic parameters on the bench *e.g.* control of ligand/template types/concentration and counter-ions could allow the development of new libraries of interesting and reliable molecular nanostructures.^12^ Despite the vast number of supramolecular clusters now known, an ongoing challenge is the understanding of the chemical relationships, interactions and molecular recognition^13^ within a given library.^14^ In a similar manner, understanding the parameters that promote the formation of increased number of structurally distinct set of inorganic building blocks will allow us to control the self-assembled structures leading to the emergence of complex architectures with the potential of intrinsic function.

Previous studies showed that the dimeric [Mo_2_O_2_S_2_]^2+^ cationic species is a very useful building block, due to inherent stability over a wide range of pH values.^2^–^4^ This is because interactions with ligands of the appropriate rigidity, charge and geometry can lead to gigantic structures. Hence, based on the above observation, the interaction of the planar and rigid {(Mo_2_O_2_S_2_)_3_(OH)_4_(C_4_O_4_)} building block with the {Mo(Mo_5_)} pentagonal unit can lead to the formation of a range of oxo(thio)metalate-based scaffolds.^4^,^15^ These scaffolds can assemble into nanomolecular architectures with nuclearities ranging from 45 to 96 metal centres, and exhibit diverse architectures.^15^–^18^ However, despite the large amount of work done and new structures discovered, the ability to use main-group oxo-anions for structural control is lacking.

**Fig. 1:**
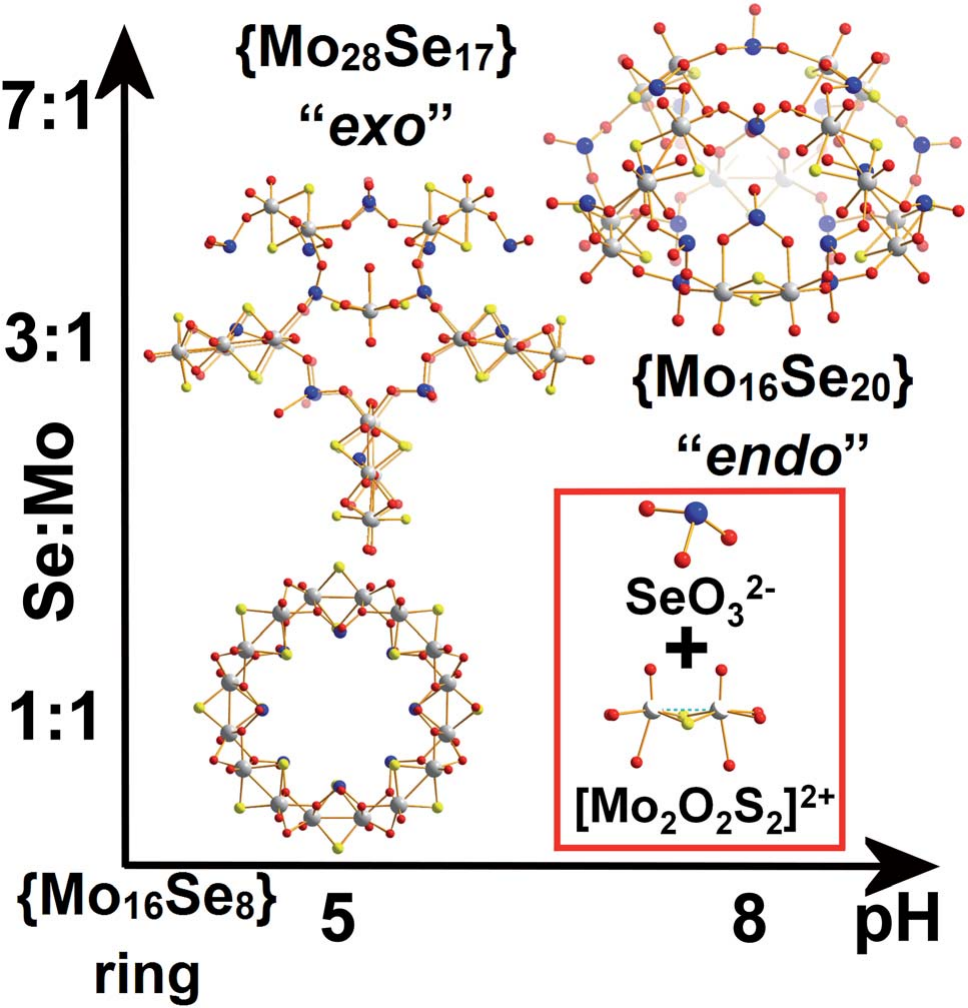
Ball-and-stick representation of the clusters “endo” {Mo_16_Se_20_} (**1**), “exo” {Mo_28_Se_17_} (**2**) and {Mo_16_Se_8_} (**3**). The gradual increase of Se concentration in the reaction mixture was reflected by the increased Se content in the isolated architectures as revealed by the X-ray diffraction analyses. Colour code: Mo, light grey; S, yellow; Se, blue; O, red.

Herein we report that control of the [Mo_2_S_2_O_2_]^2+^/SeO_3_^2–^ leads the isolation of three types of nanoscale structure; a spherical “endo” cluster, {Mo_16_Se_20_} **1**, a cross-shaped “exo” cluster, {Mo_28_Se_17_} **2**, and a ring-shaped {Mo_16_Se_8_} **3** cluster. Both compounds **1** and **2** are new members of the ChalcoPOM family (compound **3** is known),^17^ but all three compounds are accessed *via* control of the Mo : Se ratio in the reaction conditions. This has a dramatic effect appearing to trigger the formation of new sets of distinct building blocks, see Fig. 1. The two new clusters were characterized by elemental analyses, single-crystal X-ray structure analyses, bond valence sum (BVS) calculations, thermogravimetry (TGA), FT-IR and visible-NIR spectroscopy. Furthermore, we discuss the topologies, Van der Waals surface areas, proton conducting and cation recognition properties of **1** and **2** in order to explore the influence of the building block library's complexity on functionality. All the compounds discussed here can be formulated as follows:Cs_5_K_8_Na_4_H_7_[(Mo_2_O_2_S_2_)_8_(SeO_3_)_20_(H_2_O)_8_]·30H_2_O ≡ {Mo_16_Se_20_} **1**K_9_Na_9_H_7_I[(Mo_2_O_2_S_2_)_8_(SeO_3_)_20_(H_2_O)_8_]·28H_2_O ≡ {Mo_16_Se_20_} **1′**K_15_H_5_[(Mo_2_O_2_S_2_)_14_(OH)_14_(SeO_3_)_17_(H_2_O)_8_]·80H_2_O ≡ {Mo_28_Se_17_} **2**[N(CH_3_)_4_]_1.5_K_5.5_Na_2_[I_3_ ⊂ (MoV2O_2_S_2_)_8_(OH)_8_(Se^IV^O_3_)_8_]·25H_2_O **3**

## Results and discussion

The selenite anion, SeO_3_^2–^, has been known for its numerous coordination modes and used as building unit for the construction of larger architectures.^16^ With this in mind, we investigated the interaction of the redox active SeO_3_^2–^ anion with the electrophile [Mo_2_O_2_S_2_]^2+^ dimer in order to probe the interactions between the initial constituents of the system and their influence on the following assembly phases. To explore this we varied the SeO_3_^2–^ : [Mo_2_O_2_S_2_]^2+^ ratio and/or the pH value of the system leading to the formation of a variety of structures ranging from rings [N(CH_3_)_4_]_1.5_K_5.5_Na_2_[I_3_ ⊂ (MoV2O_2_S_2_)_8_(OH)_8_(Se^IV^O_3_)_8_]·25H_2_O **3** (reported in our previous work),^17^ to building block-based scaffolds K_15_H_5_[(Mo_2_O_2_S_2_)_14_(OH)_14_(SeO_3_)_17_(H_2_O)_8_]·80H_2_O **2** and nanosized hollowed-elliptical cages Cs_5_K_8_Na_4_H_7_[(Mo_2_O_2_S_2_)_8_(SeO_3_)_20_(H_2_O)_8_]·30H_2_O **1** and K_9_Na_9_H_7_I[(Mo_2_O_2_S_2_)_8_(SeO_3_)_20_(H_2_O)_8_]·28H_2_O **1′**.

**Fig. 2:**
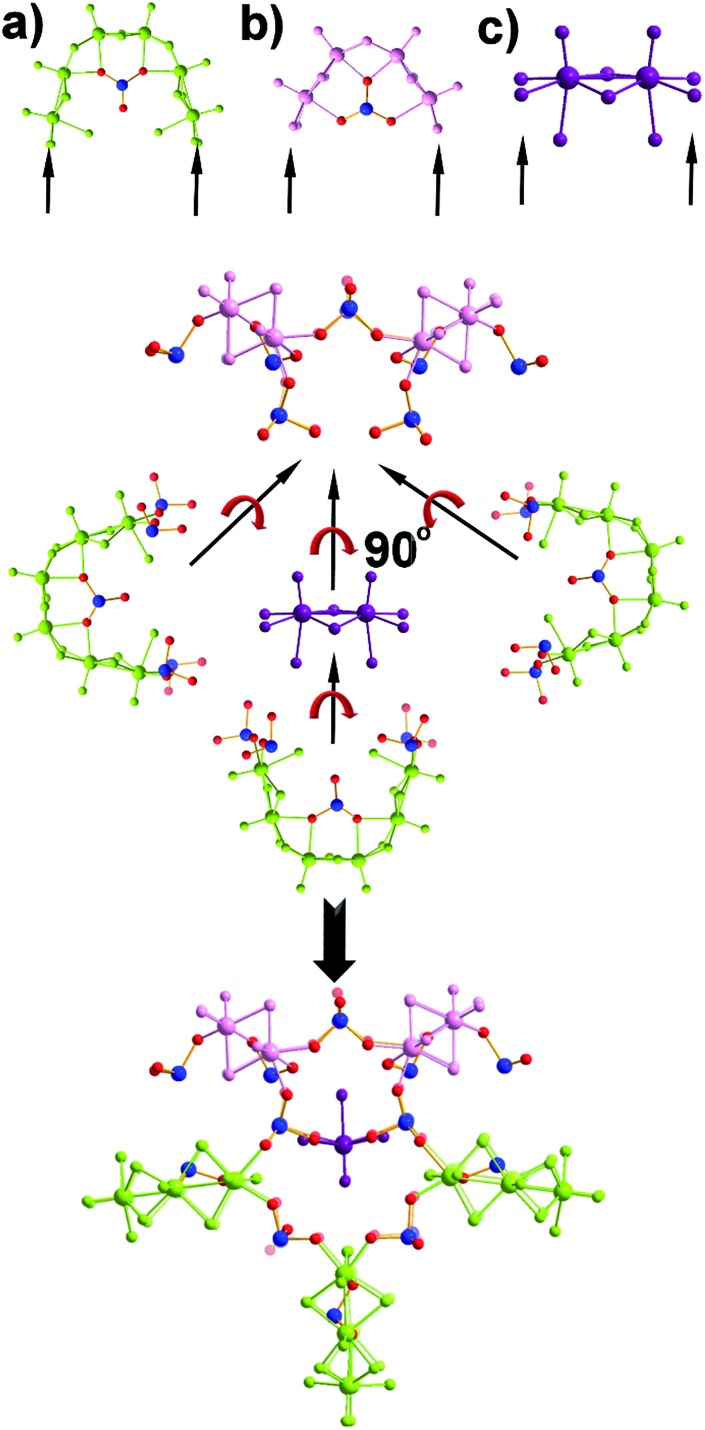
Representation of the structure of the {Mo_28_Se_17_} (compound **2**). Generation of the (a) [(Mo_2_O_2_S_2_)_3_(OH)_4_(H_2_O)_6_(SeO_3_)], (b) [(Mo_2_O_2_S_2_)_2_(OH)_2_(H_2_O)_4_(SeO_3_)] and (c) [Mo_2_O_2_S_2_(H_2_O)_6_]^2+^ building blocks. The templating role of the dimeric building block (c) allows the connection of three (a)- and two (b)-type building blocks orthogonal to the plane defined by the architecture.

The reaction between SeO_3_^2–^ and [Mo_2_O_2_S_2_]^2+^ at 1 : 1 ratio at pH = 5.0–5.5, produced a wheel shaped cluster **3** with the tendency to form tubular structures in the solid state.^17^ When a ratio of 3 : 1 was used instead, an “exo” type architecture was isolated constructed by three types of distinct building blocks, Fig. 2. An intriguing observation is that compounds **2** and **3** were obtained within the same range of pH values *ca.* 5.0–5.5 using the same initial constituents. However, the relevant ratio of these components changed the interaction between SeO_3_^2–^ and [Mo_2_O_2_S_2_]^2+^ leading to increased number of distinct building blocks formed into the solution which can assemble into architectures of increased complexity and considerably different topology (Fig. 1). When the amount of SeO_3_^2–^ increased further in the reaction mixture to a SeO_3_^2–^ : [Mo_2_O_2_S_2_]^2+^ ratio of 7 : 1 and the pH of the solution adjusted at *ca.* 8, compound **1** was isolated from the reaction mixture. In the latter case, the anionic porous molecular cage with “endo” architecture [(Mo_2_O_2_S_2_)_8_(SeO_3_)_20_(H_2_O)_8_]^24–^**1′a** was obtained initially as potassium salt K_9_Na_9_H_7_I[**1′**]·36H_2_O which crystallized in a highly symmetrical cell lattice. Interestingly, addition of CsCl in the reaction mixture led to the formation of an isostructural molecular cage where in this case showed site selective recognition for Cs^+^ cations. Also in this case, we observed a dramatic influence on the interactions between the initial constituents of the reaction mixture. The modification of the Se : Mo ratio of the reaction, give rise to the generation of markedly different building block library which was reflected further on the modulation of the topology from wheel and “exo” to “endo” type molecular cage.

The first effort to investigate the influence of the selenite anions on the self-condensation of [Mo_2_O_2_S_2_]^2+^ cation led to the formation of the ring type cluster {Mo_16_Se_8_} **3**.^17^ When the amount of the selenite anion was gradually increased, the new cluster **2** was isolated as orange rod shaped single crystals in space group *P*1[combining macron]. X-ray single-crystal analysis revealed a complex “exo” structural motif constructed by three different types of distinct building blocks: (a) [(Mo_2_O_2_S_2_)_3_(OH)_4_(SeO_3_)(H_2_O)_6_] ≡ {Mo_6_}, (b) [(Mo_2_O_2_S_2_)_2_(OH)_2_(H_2_O)_4_(SeO_3_)] ≡ {Mo_4_} and (c) [Mo_2_O_2_S_2_(H_2_O)_6_]^2+^ ≡ {Mo_2_} (Fig. 2 and S1†). The SeO_3_^2–^ anion templates the formation of two new building blocks, [(Mo_2_O_2_S_2_)_3_(OH)_4_(SeO_3_)(H_2_O)_6_] and [(Mo_2_O_2_S_2_)_2_(OH)_2_(H_2_O)_4_(SeO_3_)], exhibiting two different coordination modes (Fig. 2 and S2†). The upper part consists of a ring shaped formation {[(Mo_2_O_2_S_2_)_2_(OH)(SeO_3_)]_2_(SeO_3_)_4_}^6–^, where the selenite anions exhibit a plethora of coordination modes including monodentate (η^2^-), bidentate (μ-) and tridentate (μ_3_-, μ_4_-) (Fig. S1b†); the middle synthon is composed of one [Mo_2_O_2_S_2_]^2+^ unit and four SeO_3_^2–^ anions which templates and stabilizes the formation of the “exo” architecture by linking the upper and lower part consisting of three [(Mo_2_O_2_S_2_)_3_(OH)_4_(SeO_3_)(H_2_O)_6_] building blocks linked by four μ-SeO_3_^2–^ anions (Fig. S1d†). The selenite anion exhibits a dual role in this chemical system since not only acts as an effective anionic ligand by linking together the generated building blocks but also templates the formation of two novel building blocks, {Mo_6_} and {Mo_4_}, used for the construction of the nanosized “exo” architecture. Alternatively, the compound **1** is constructed by 3 × {Mo_6_} and 2 × {Mo_4_} building blocks placed approximately 6 Å apart forming an angle of approximately 64° around a central [Mo_2_O_2_S_2_]^2+^ template (Fig. 2). Due to the connectivity of the building blocks, the overall symmetry of the architecture is decreased to *C*_s_ since there is no proper rotation axis.

**Fig. 3:**
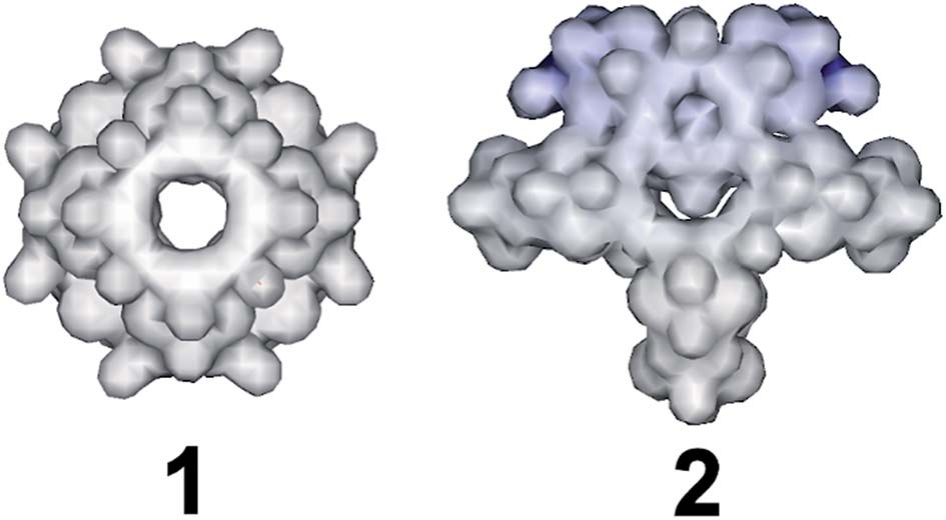
Representation of the Van der Waals surface areas of the “endo” (compound **1**/**1′**) and “exo” (compound **2**) structures. The relative surfaces areas found to be 1694 and 2145 Å^2^ respectively.

When the amount of sodium selenite is increased significantly while adjusting the molar ratio of SeO_3_^2–^ : [Mo_2_O_2_S_2_]^2+^ in the reaction mixture to a value of *ca.* 7 : 1, the assembly of the building blocks was directed towards the formation of an “endo” structural motif, at high pH (*ca.* 8.0), forming an open nanostructured-cage, compound **1**, Fig. 1a. The higher concentration of the selenite anion in the reaction mixture favoured the generation of different kinetically stable building blocks with a curved structure (see ESI†) that introduce curvature to the final architecture leading to the formation of the ellipsoid “endo” topology observed in compound **1**. The “endo” and “exo” structural features can be quantified directly by measuring the Van der Waals surface areas which are found to be *ca.* 1694 and 2145 Å^2^ for the “endo” (**1**/**1′**) and “exo” (**2**) architecture respectively, Fig. 3. The assembly of the selenite templated building blocks led to the formation of an extended molecular structure exhibiting an “exo” topology **2**, (at lower selenite concentrations). In this case, the total surface area of the assembled molecular “panels”, Fig. 2, is higher, 2145 Å^2^, than the ellipsoidal structure with “endo” topology **1**, with smaller space requirements and total outer surface of 1694 Å^2^. This observation shows that it is synthetically possible to control the architecture formed *i.e.* ring, “endo” or “exo” structural motif and this is directly reflected on the number of selenite anions incorporated in the final structure. The relatively high pH of the reaction mixture and SeO_3_^2–^ : [Mo_2_O_2_S_2_]^2+^ ratio, appears to facilitate the ability of SeO_3_^2–^ anion to act as a ligand rather than template, linking predominantly the [Mo_2_O_2_S_2_]^2+^ building blocks into a {Mo_16_Se_20_} cage-structure. X-ray diffraction analysis revealed an open cage architecture which consists of eight [Mo_2_O_2_S_2_]^2+^ dimer-units linked by 20 selenite ligands exhibiting an idealised *C*_4v_ symmetry. All the selenite anions in this cluster adopt the bidentate (μ-) coordination mode. The structure has an idealized *C*_4_ rotation axis and elliptical porous architecture of 17.1 × 10.3 Å; each quarter consists of three [Mo_2_O_2_S_2_]^2+^ cations and six SeO_3_^2–^ anions which link the neighbouring [Mo_2_O_2_S_2_]^2+^ moieties (Fig. S3†). These four quarters are linked further together *via* Mo–O bonds [1.98(5)–2.11(8) Å] forming a cage with three different types of windows and two different types of anchor points within the cavity.

**Fig. 4:**
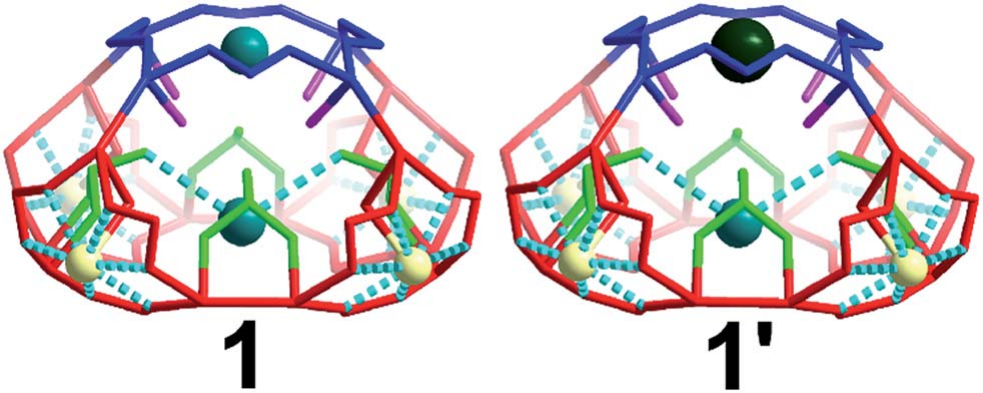
Representation of the different pores and internal anchor points in compound **1** and **1′**. The different types of pores and anchor points are highlighted along with their dimensions (Å): blue (6.6); red (5.3); pink (5.6); green (6.1). Na, light yellow; K, teal; Cs, dark green.

There are four windows located on the side of the cage (highlighted red) and one on the top of the architecture (highlighted blue) of 5.4 and 6.6 Å respectively (Fig. 4). Additionally, there are two different types of oxygen anchor points within the cavity (highlighted in green and pink) located at a distance of 5.5 and 6.1 Å respectively. Consequently, the cage is able to act as site selective inorganic cryptand and accommodate cations based on their atomic radius. In our first synthetic effort to isolate **1** in the presence of Na^+^ and K^+^ cations, the red coloured pores were occupied by Na^+^ cations whilst blue coloured pores and the internal anchor point formed by selenite anions (green) occupied by K^+^ cations, Fig. 4. Interestingly, in the presence of Na^+^, K^+^ and Cs^+^ we observed that the red pores (5.4 Å) and internal anchor points (5.5 Å) were occupied by the same type of cations but the blue coloured pore (6.6 Å) showed preferential cation recognition behaviour by capturing Cs^+^ from the mixture of cations instead of K^+^. This is a rare example of multifunctional inorganic cryptand with multiple cation binding sites.

Based on our previous observation that ChalcoPOMs are promising proton conductors, we opted to investigate the relevant property of our clusters using alternating-current (AC) impedance measurements. The measurements were conducted on pellet samples in a range of relative humidity environment. The log(*σ*) (S cm^–1^) *versus* RH% (relative humidity) profiles at 20 °C are shown in Fig. S13.† The proton conductivities were determined from the obtained Nyquist plots. The low-frequency tail observed in the Nyquist plots (Fig. S11 and S12†) is consistent with blocking effects at the electrode, as would be expected for ionic conduction. The proton conductivity at 20 °C for **1** and **2** at 33% RH are 2.7 × 10^–6^, and 4.0 × 10^–7^ S cm^–1^, respectively whilst at 97% RH are 2.0 × 10^–3^ and 2.4 × 10^–4^ S cm^–1^.

**Fig. 5:**
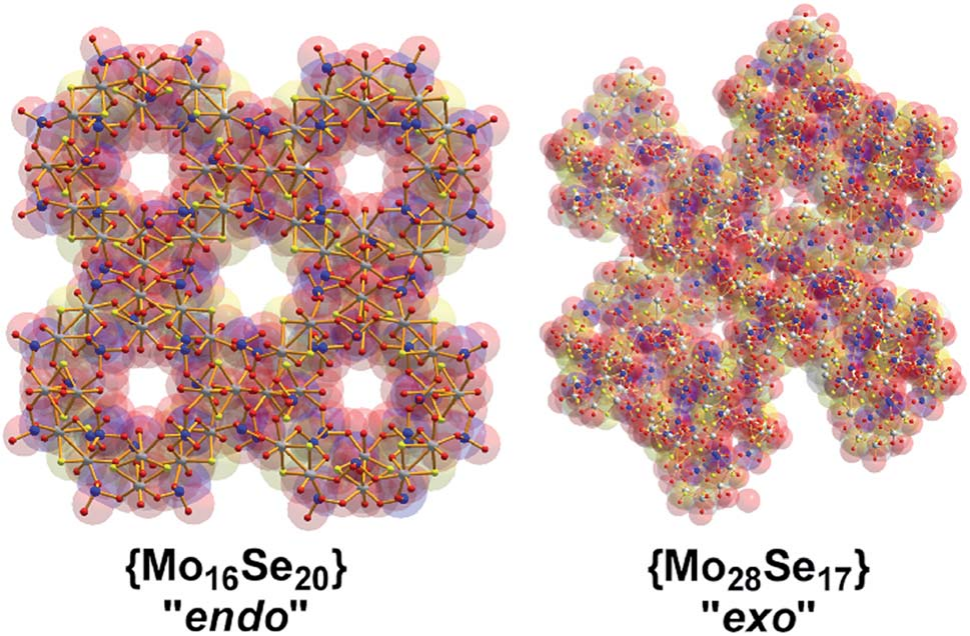
Representation of the packing motifs of {Mo_16_Se_20_} and {Mo_28_Se_17_} along *c* axis. The intermolecular interactions in {Mo_16_Se_20_} **1**/**1′** “engineer” channels of 5.2 and 7.1 Å respectively whilst the closely packing of {Mo_28_Se_17_} **2** clusters minimize the size of the available pathways.

Compound **1** exhibits a better conductivity than **2** at the same experimental conditions since **1** incorporates twenty available oxo-groups from the coordinated selenite anions which can act as effective proton carriers. These, in combination with the channels along *c* axis, appears to provide an efficient proton conduction pathway (Fig. S4†).^25^,^26^ Compound **2** has less available oxo (Se–O) groups, whilst the clusters are tightly packed in the solid state reducing the available conduction pathways, Fig. 5. Indeed, the conductivity values recorded appear to be improved compared with MOF-based materials which were recently reported^26^ with values ranging from 2 × 10^–9^ to 10^–3^ S cm^–1^.

## Conclusions

To conclude, we demonstrate here a new type of assembly which is crucial for the regulation of the intermolecular interactions between the primary constituents of the SeO_3_^2–^/[Mo_2_O_2_S_2_]^2+^ chemical system. The alteration of the chemical species' populations induces diversity to the generated building block library which can subsequently assemble into complex architectures. The assembly of the primary constituents is the key factor that influence the final structural and physical properties of the isolated clusters. This behaviour greatly depends on the coordinative ability of the selenite anions allowing the easy modulation of the interactions within the [Mo_2_O_2_S_2_]^2+^/SeO_3_^2–^ system, leading to the generation of new ChalcoPOM based building blocks. The increase of the structurally distinct components in the chemical system seems to influence its complexity which allowed topological control and potential correlation with the observed properties. In this case, we observed the generation of two new building blocks, namely: [(Mo_2_O_2_S_2_)_3_(OH)_4_(SeO_3_)(H_2_O)_6_] ≡ {Mo_6_}, [(Mo_2_O_2_S_2_)_2_(OH)_2_(H_2_O)_4_(SeO_3_)] ≡ {Mo_4_}. The assembly stage, directed the organization of the building blocks, into nanostructured molecular species **1**, **2** and **3** which exhibit “exo”, “endo” and ring structural motifs. In the case of the “endo” structural motif **1**, X-ray diffraction analysis revealed an elliptical porous molecular cage exhibiting a Van der Waals surface area of 1694 Å^2^ considerably smaller to the one observed in the case of “exo” **2** structure (2145 Å^2^). Moreover, cluster **2** is a rare example of inorganic cryptand which exhibits multiple cation binding sites. Thus the assembly stage of the primary constituents along with the versatility of the SeO_3_^2–^ anions allowed us to modulate the final topologies of the clusters (ring *vs.* “endo” *vs.* “exo”). Furthermore, the compounds reported are found to be promising proton conductors with 2.4 × 10^–4^ and 2.0 × 10^–3^ S cm^–1^ (RH = 97% at 20 °C). As such, the gradual increase of the selenium content in the architectures led to more available proton carrier points (Se–O) in combination with the open structures (compound **1** and **2**). Overall, we showed that the increase of the number of structurally distinct components in the chemical system, increased the complexity of the formed species which is associated with the observed functionality.

Future work will focus our efforts on investigating further the potential of these new processes and biology inspired principles in inorganic systems since increase of diversity induces the generation of new building block libraries, increase the system's structural complexity and identification of the correlation between structure and observed functionality.

## Experimental

### Materials and instrumentation

All reagents and chemicals were purchased from Sigma Aldrich Chemical Company Ltd. and Alfa Aesar. Unless stated otherwise, the materials were used without further purification. The dimeric [Mo_2_S_2_O_2_]^2+^ unit was synthesized according to the modified published procedure by E. Cadot *et al.*^19^ and the [Mo_2_O_2_S_2_(H_2_O)_6_]^2+^ solution obtained was stored under Ar. The [N(CH_3_)_4_]_1.5_K_5.5_Na_2_[I_3_ ⊂ (MoV2O_2_S_2_)_8_(OH)_8_(Se^IV^O_3_)_8_]·25H_2_O (**3**) cluster was prepared according to the previously reported procedure.7*a* Flame atomic absorption spectroscopy (FAAS) and CHN elemental analyses were performed at the Environmental Chemistry Section and microanalysis services within the School of Chemistry, on a Perkin-Elmer 1100B Atomic Absorption Spectrophotometer and an EA 1110 CHN, CE-440 Elemental Analyser, respectively. Thermogravimetric analysis was performed on a TA Instruments Q 500 Thermogravimetric Analyzer under nitrogen flow at a typical heating rate of 5 °C min^–1^. UV-Vis spectra were collected using a Shimadzu PharmaSpec UV-1700 UV-Vis spectrophotometer in transmission mode using quartz cuvettes with 1.0 cm optical path length. Infrared spectra (4000–400 cm^–1^) of all samples were recorded on JASCO FTIR-410 spectrometer or a JASCO FT-IR 4100 spectrometer. Characteristic IR bands are shown in cm^–1^; intensities denoted as s = strong, m = medium, w = weak, sh = sharp.

### Synthesis of compound {Se_16_Mo_20_} = Cs_5_K_8_Na_4_H_7_[(Mo_2_O_2_S_2_)_8_(SeO_3_)_20_(H_2_O)_8_]·30H_2_O (**1**)

Na_2_SeO_3_ (1.22 g, 7 mmol) was dissolved in 10 mL of deionized water. Then 7.4 mL (1.0 mmol) of the dimmeric [Mo_2_O_2_S_2_]^2+^ starting material was diluted with 10 mL of deionized water and added into the above solution (pH ∼ 8). Then CsCl (0.17 g, 1.0 mmol) was added into the reaction mixture forming a yellow precipitate. The solution was stirred under nitrogen atmosphere at room temperature for about 1 hour. The solution was filtered and the filtrate left undisturbed in a conical flask sealed with pierced parafilm at 18 °C. Orange red block shaped crystals formed after a period of 22 weeks. Yield: 360 mg (43.7% based on Mo^V^). Elemental analysis for **1** (H_83_Cs_5_K_8_Na_4_Mo_16_O_114_S_16_Se_20_, Fw. 6604.17 g mol^–1^); Cal.: H: 1.27; Cs: 10.06; K: 4.74; Mo: 23.24; Na: 1.39; S: 7.77; Se: 23.91; found: H: 1.14; Cs: 10.82; K: 4.94; Mo: 22.55; Na: 1.21; S: 6.53; Se: 23.44. IR (KBr, cm^–1^): 3379.6 (s, broad) [–OH]; 1633.4 (m) [H_2_O]; 1120.4 (w); 937.2 (m) [Mo=O]; 850.12 (m) [Se–O]; 707.7 (sh) [Mo–OH–Mo]; 531.12 (w) [Mo–S–Mo].

### Synthesis of compound {Se_16_Mo_20_} = K_9_Na_9_H_7_I[(Mo_2_O_2_S_2_)_8_(SeO_3_)_20_(H_2_O)_8_]·28H_2_O (**1′**)

The cluster K_9_Na_9_H_7_I[(Mo_2_O_2_S_2_)_8_(SeO_3_)_20_(H_2_O)_8_]·28H_2_O (**1′**) was synthesized following the synthetic procedure for **1** in the absence of CsCl. Elemental analysis for **1′** (H_79_IK_9_Na_9_Mo_16_O_112_S_16_Se_20_, Fw. 6184.6 g mol^–1^); Cal.: H: 1.29; K: 5.69; Na: 3.35; Mo: 24.82; S: 8.29; Se: 25.53; found: H: 1.21; K: 4.94; Na: 3.12; Mo: 25.55; S: 7.72; Se: 24.58.

### Synthesis of compound {Se_17_Mo_28_} = K_15_H_5_[(Mo_2_O_2_S_2_)_14_(OH)_14_(SeO_3_)_17_(H_2_O)_8_]·80H_2_O (**2**)

7.4 mL (1.0 mmol) of the dimmer [Mo_2_O_2_S_2_]^2+^ solution was diluted with 10 mL of deionized water. The pH of the solution was adjusted to 5.5 with 1 M K_2_CO_3_. Na_2_SeO_3_ (0.52 g, 3.0 mmol) dissolved in 20 mL of deionized water and subsequently added into the above solution forming a yellow mixture with final pH value of 8.7. Then the pH of the obtained solution was re-adjusted to 5.0–5.5 (maximum yield at pH = 5.5) by addition of HAc (60%) and the final reaction mixture was stirred at room temperature for one hour. The colour of the solution changed gradually to orange-red while a small amount of unidentified solid precipitated. The precipitate was filtered off and the reaction mixture left undisturbed in a 50 mL beaker at 18 °C. Orange block shaped crystals formed after a period of 10 weeks. Yield: 350 mg (56.8% based on Mo^V^). Elemental analysis for **2** (H_195_K_15_Mo_28_O_181_S_28_Se_17_, Fw: 8605.4 g mol^–1^); Cal.: H: 2.28; K: 6.81; Mo: 31.22; S: 10.43; Se: 15.60; found: H: 1.98; K: 6.44; Mo: 31.26; S: 11.05; Se: 15.97. IR (KBr, cm^–1^): 3375.8 (s, broad) [–OH]; 1616.1 (m) [H_2_O]; 943.0 (sh) [Mo=O]; 849.77 (m) [Se–O]; 717.4 (sh) [Mo–OH–Mo]; 518.8 (m) [Mo–S–Mo].

### X-ray crystal structure analyses

Suitable single crystal was selected and mounted onto a rubber loop using Fomblin oil. Single-crystal X-ray diffraction data of **1**, **1′** and **2** were recorded on a Bruker Apex CCD diffractometer (*λ* (Mo Kα) = 0.71073 Å) at 150 K equipped with a graphite monochromator. Structure solution and refinement were carried out with SHELXS-97^20^ and SHELXL-97^21^ using the WinGX software package.^22^ Data collection and reduction were performed using the Apex2 software package. Corrections for incident and diffracted beam absorption effects were applied using empirical absorption corrections. All the Mo atoms (including those disordered) and most of the O atoms were refined anisotropically. Solvent water molecule sites with partial occupancy were found and included in the refinement of the structure. Crystallographic formulae typically contain a lot more water molecules in the crystal lattice than the formulae used for chemical analyses as the sample was dried up. It is important to note that with these structures we are moving outside the realm of small molecule crystallography and are dealing with refinements and problems that lie between small molecule and protein crystallography. As a result we cannot expect refinements and statistics to follow the path of crystals with much smaller unit cells. However, the final refinement statistics are good and in all cases the structural analysis allows us to unambiguously determine the structures of the reported clusters. Crystallographic data for compounds **1**, **1′** and **2** (CSD 428785, 430164 and 428786).

### Proton conductivity

Pellets of 1.3 cm in diameter were pressed at 1000 kg N for 2 min using standard IR dies and sandwiched between two gold-coated steel electrodes. The proton conductivities of all the samples were measured using the two – probe method conductivity cells by AC impedance spectroscopy technique. The samples were placed in several closed chambers with different relative humidity (RH) environments using a range of standard saturated salt solutions, MgCl_2_ (∼33% RH), (Mg(NO_3_)_2_) (∼53% RH), NaNO_2_ (∼65% RH), NaCl (∼75% RH) and K_2_SO_4_ (∼97% RH).^23^ The samples were placed and remained in the humidity chamber for 3 days to ensure that the air in the bottle reached to equilibrium state in our investigations. The measurements were performed using a multi-channel potentiostat VMP3 by Bio-logic Instruments over the frequency range 1 Hz to 1 MHz with input voltage amplitude of 20 mV. ZView software was used to fit impedance data sets by means of an equivalent circuit simulation to obtain the resistance values. The conductivity (*σ*, S cm^–1^) of the sample was calculated from the impedance data, using the formula *σ* = *L*/*RA*, where *L* (cm) is the thickness of the sample, *A* (cm^2^) is the face area and *R* (Ω) is the sample's resistance estimated by extrapolation of the high frequency arc crossing to the real axis.^24^ The measurements have been repeated three times.

## Supplementary Material

Supplementary information
