# Supplementary material for: Assembly of inorganic [Mo2S2O2]2+ panels connected by selenite anions to nanoscale chalcogenide–polyoxometalate clusters
Source: Chem Sci. 2016 Feb 25;7(6):3798–804. doi: 10.1039/c5sc04944j (PMC6013829; doi:10.1039/c5sc04944j)
Supplement: Supplementary file 1 [file SC-007-C5SC04944J-s001.pdf]

Electronic Supplementary Material (ESI) for Chemical Science.
This journal is © The Royal Society of Chemistry 2016

Supporting Information

Assembly of Inorganic $[Mo_2S_2O_2]^{2+}$ Panels Connected by Selenite anions to Nanoscale Chalcogenide-Polyoxometalate Clusters

Hong-Ying Zang, Jia-Jia Chen, De-Liang Long, Leroy Cronin* and Haralampos N. Miras*

WestCHEM, School of Chemistry, The University of Glasgow, Glasgow, G12 8QQ, U.K.

Corresponding Author

charalampos.moiras@glasgow.ac.uk; lee.cronin@glasgow.ac.uk

1. Crystallography

Table S1 X-ray crystallographic data and structure refinement for compounds **1** and **2**.

**Value**	**1**	**1'**	**2**
Formula	$H_{83}Cs_5K_8Na_4Mo_{16}O_{114}S_{16}Se_{20}$	$H_{79}IK_9Na_9Mo_{16}O_{112}S_{16}Se_{20}$	$H_{195}K_{15}Mo_{28}O_{181}S_{28}Se_{17}$
M_r g mol^{-1}	6604.17	6184.54	8605.38
crystal system	Tetragonal	Tetragonal	Triclinic
space group	*P*-42(1)*m*	*P*4/*nmm*	*P*-1
a [Å]	30.7159(7)	25.5702(7)	22.4356(10)
b [Å]	30.7159(7)	25.5702(7)	23.4055(10)
c [Å]	20.0056(6)	28.9646(10)	23.6633(10)
α [˚]	90	90	103.291(2)
β [˚]	90	90	108.421(2)
γ [˚]	90	90	98.352(2)
ρ_{cald} [g cm^{-3}]	2.320	2.169	2.563
V [Å^3]	18874.6(10)	18938.1(12)	11149.4(8)
Z	4	4	2
$\mu(Mo_{K\alpha})$mm^{-1}	6.313	5.505	4.942
T [K]	150	150	150
no. rflns (measd)	117659	133578	134465
no. rflns (unique)	14851	9093	43714
no. params	861	5976	2247
Goodness-of-fit on F^2	0.965	1.089	1.079
*R*1 (I>2σ(I))	0.0879	0.0644	0.0652
*wR*2 (all data)	0.2591	0.2533	0.2155

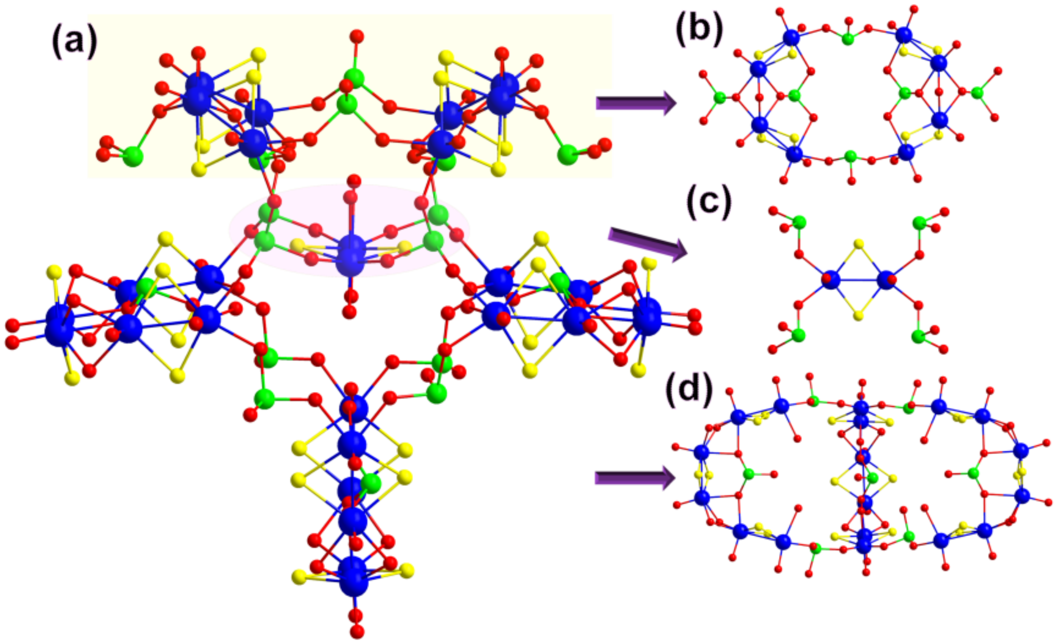

Figure S1 Ball-and-stick representation of **2** {$Se_{17}Mo_{28}$} (a) and the component parts of the structure inset (b), (c) and (d).

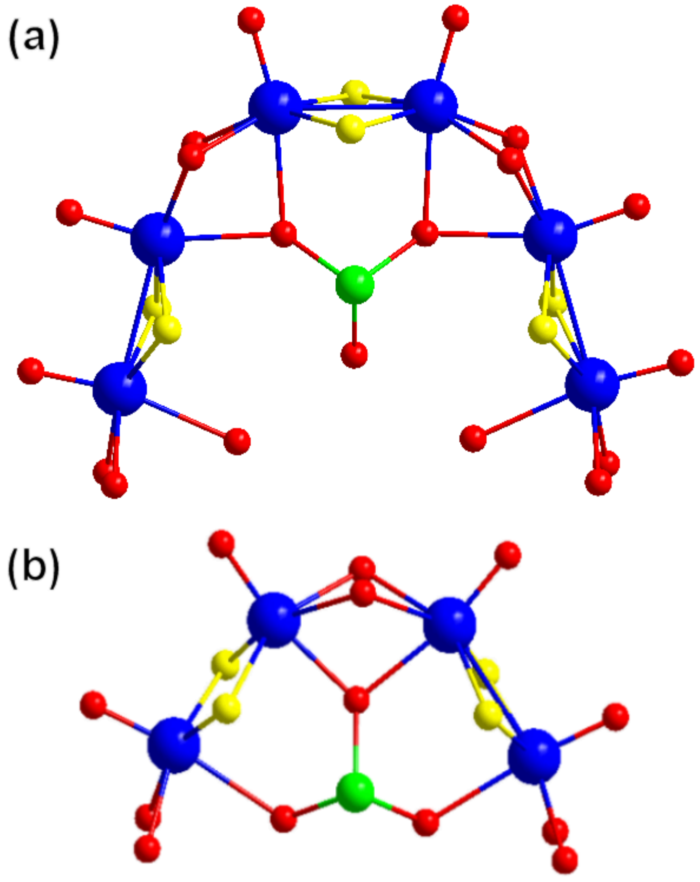

Figure S2 SeO_3^{2-} templates the self-condensation of $[Mo_2O_2S_2]^{2+}$ thiometalate unit to form the following building blocks: (a) $[(Mo_2O_2S_2)_3(OH)_4(SeO_3)]$ and (b) $[(Mo_2O_2S_2)_2(OH)O(SeO_3)]$ (Mo: Blue; S: yellow; Se: Green; O: Red).

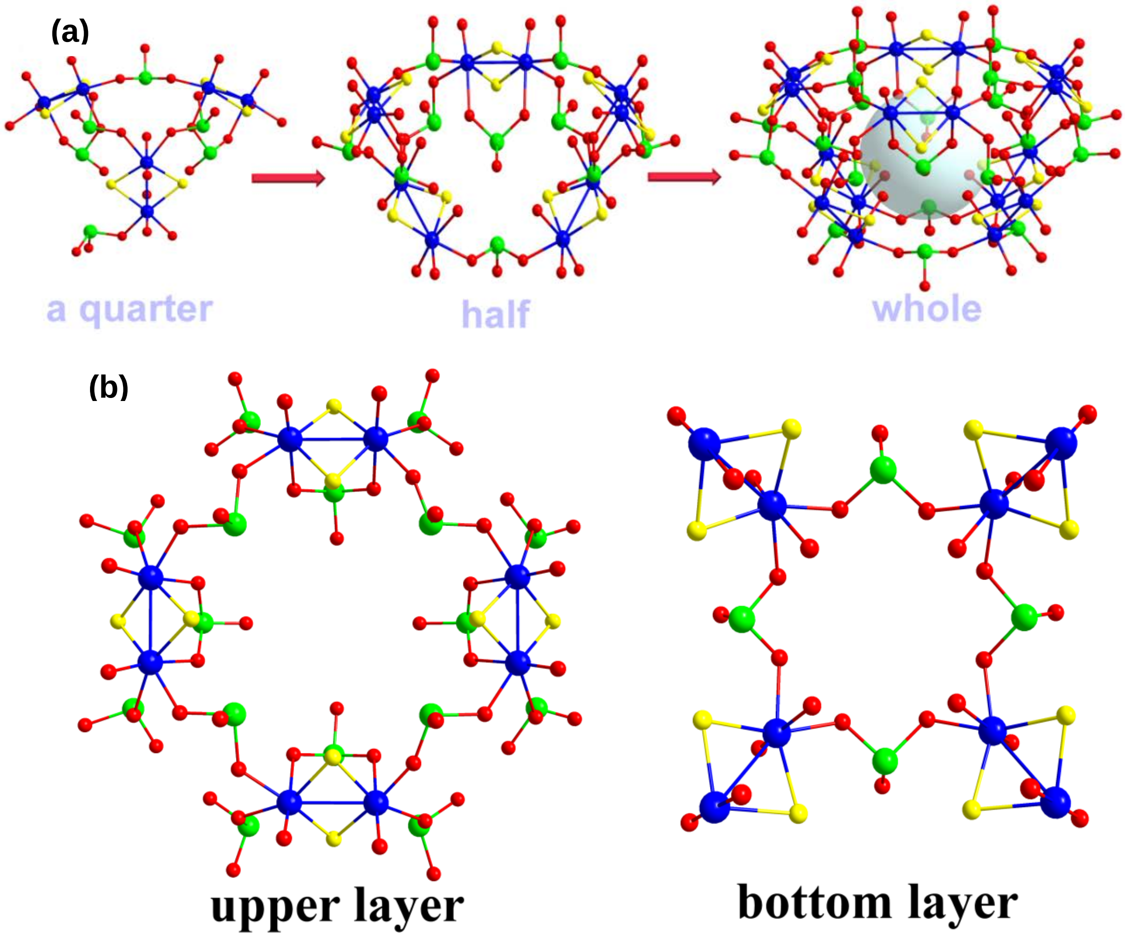

Figure S3 Ball-and-stick representation of **1** {$Se_{20}Mo_{16}$}: (a) The structure has an idealized C_4 symmetry axis and is formed by the $[(Mo_2O_2S_2)_3(SeO_3)_6]^{6-}$ repeating unit; (b) The projection view of two divided parts- the upper layer and bottom layer.

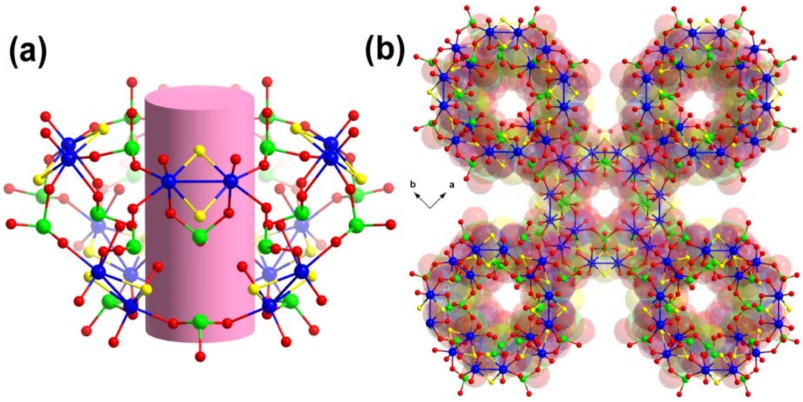

Figure S4 (a) The cavity channel in the cage **1**; (b) Packing diagram of **1** along *c* axis.

2. Fourier-transform infrared (FT-IR) spectroscopy.

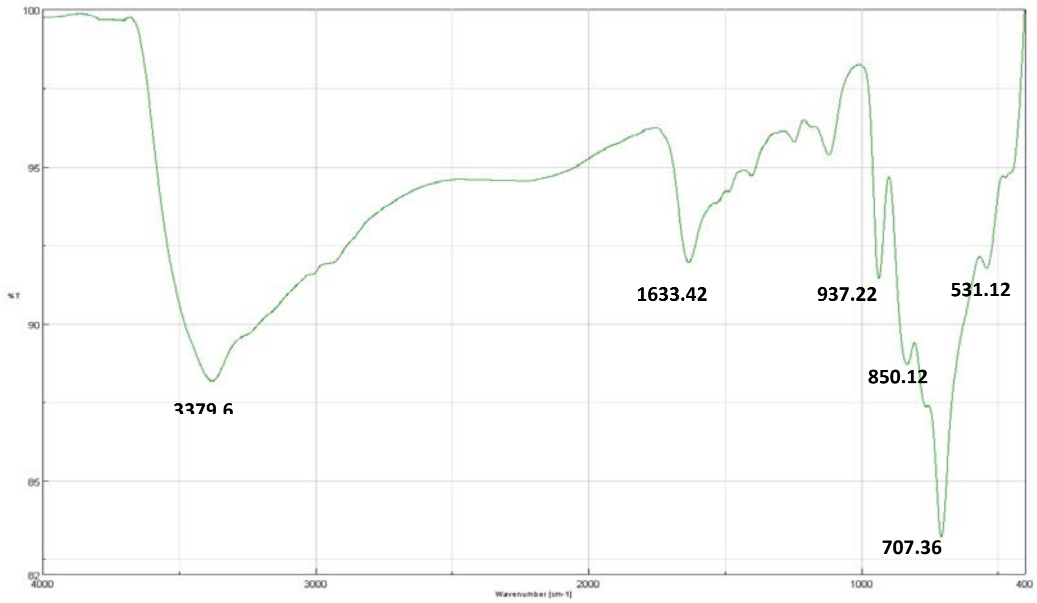

Figure S5. FT-IR (KBr pellet) spectrum for compound **1** $\{Se_{16}Mo_{20}\}$. 3379.6 (s, broad) [-OH]; 1633.4 (m) [H_2O]; 1120.4 (w); 937.2 (m) [Mo=O]; 850.12 (m) [Se-O]; 707.7 (sh) [Mo-OH-Mo]; 531.12 (w) [Mo-S-Mo].

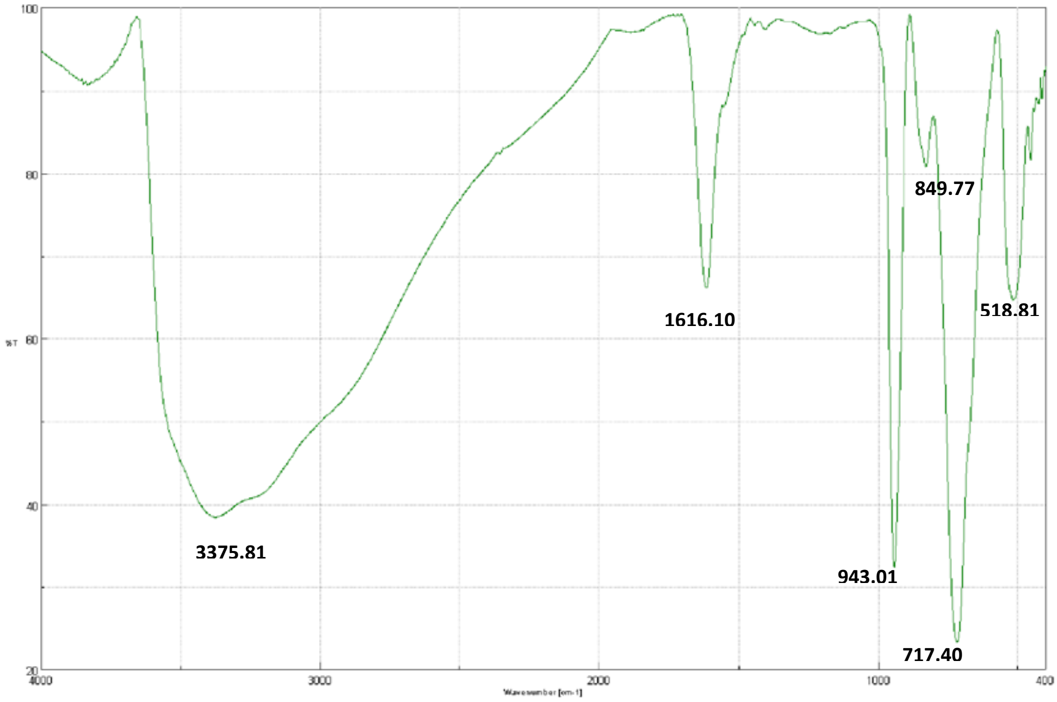

Figure S6. FT-IR (KBr pellet) spectrum for compound **2** $\{Se_{17}Mo_{28}\}$. 3375.8 (s, broad) [-OH]; 1616.1 (m) [H_2O]; 943.0 (sh) [Mo=O]; 849.77 (m) [Se-O]; 717.4 (sh) [Mo-OH-Mo]; 518.8 (m) [Mo-S-Mo].

3. UV-Vis spectroscopy

According to the UV-vis studies, compounds **1** and **2** retain their structural integrity in aqueous medium at pH values higher than 5. The recorded UV-vis spectra as a function of the time are shown in Figures S7 and S8 for compounds **1** and **2** respectively, where it's clear that both compounds are stable for at least 24 hours.

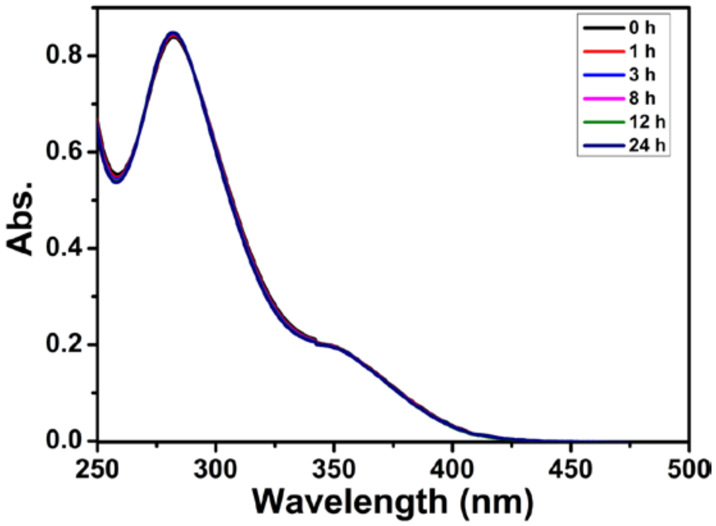

Figure S7. UV-vis spectrum for compound **1** {$Se_{16}Mo_{20}$} in water.

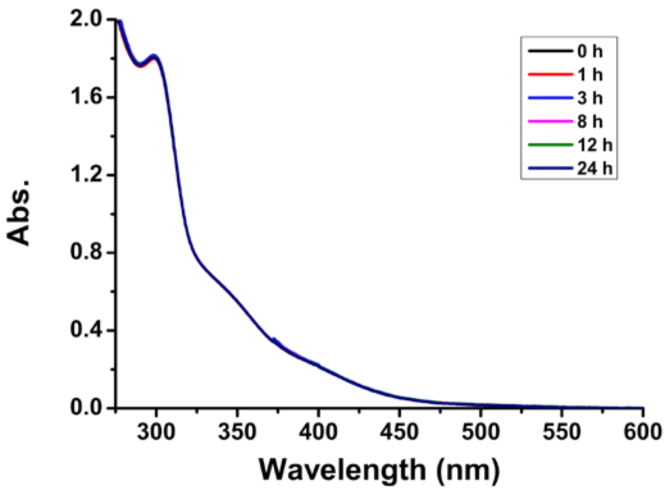

Figure S8. UV-vis spectrum for compound **2** {$Se_{17}Mo_{28}$} in water.

4. Thermogravimetric analysis (TGA)

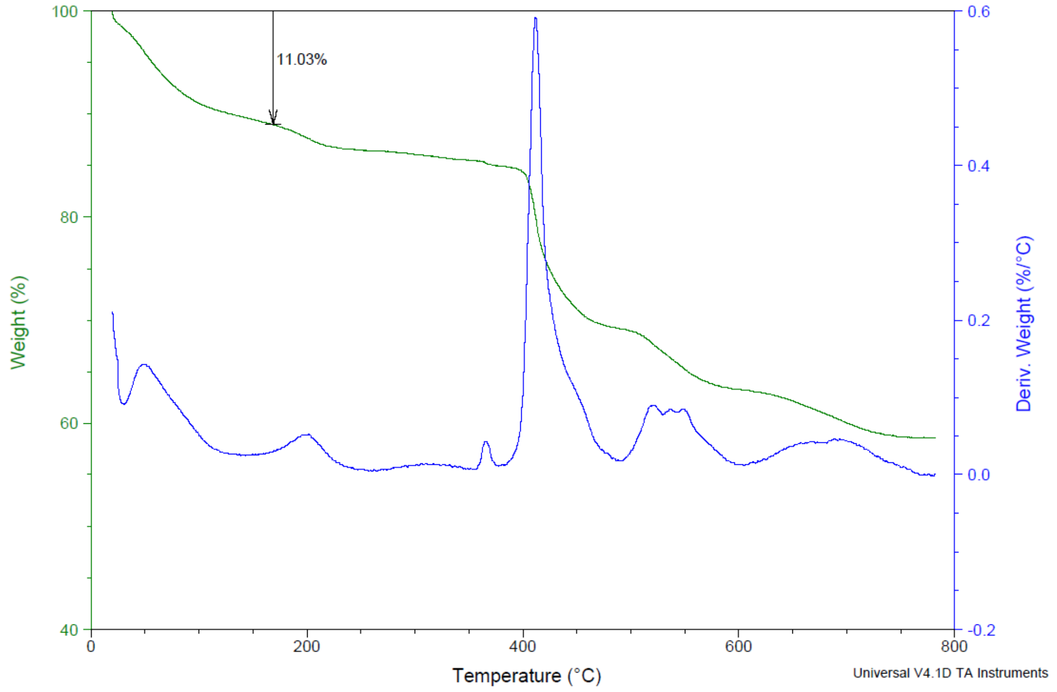

Figure S9. Thermal gravimetric analysis of **1** showing the loss of solvent content (RT – 170 ºC) followed by the elimination of the sulphur content in the form of SO_2 and subsequent decomposition of the framework.

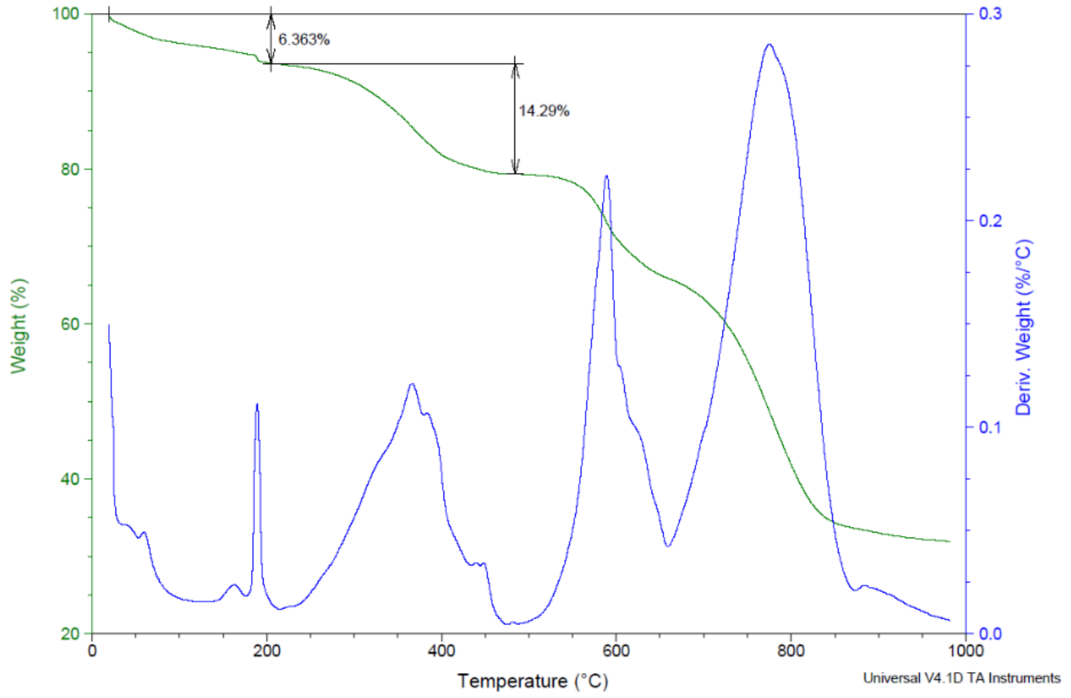

Figure S10. Thermal gravimetric analysis of **2** showing the loss of solvent content (RT – 190 ºC) followed by the elimination of the sulphur content in the form of SO_2 (300 – 800 °C) and subsequent decomposition of the framework.

5. Proton conductivity

The conductivity is typically derived from the "semi-circle"-shaped high frequency region of the impedance data while the slope which falls within the low frequency region is linked with the diffusion of cations within the electrodes and associated with the Warburg resistance. Very often, the testing system and environmental/experimental variables influence the Warburg diffusion. It is generally acceptable and reasonable that part of the Warburg slope is not perfectly overlapped with the simulated one due to the fact that the actual system under investigation cannot be 100% equivalent within a wide range of frequencies to the ideal simulated circuit. The precisely simulated results for the semi-circle part of the graph though gives accurate and reliable conductivity data.

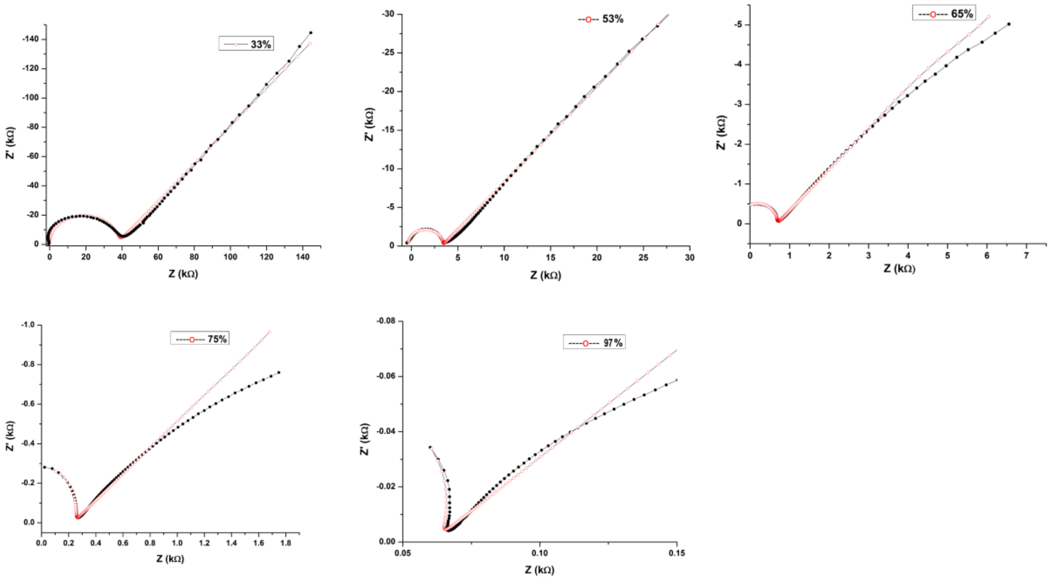

Figure S11. Nyquist plots for compound **1** at various relative humidity values at 20 °C; black dots are experimental data and red hollow dots are simulated data.

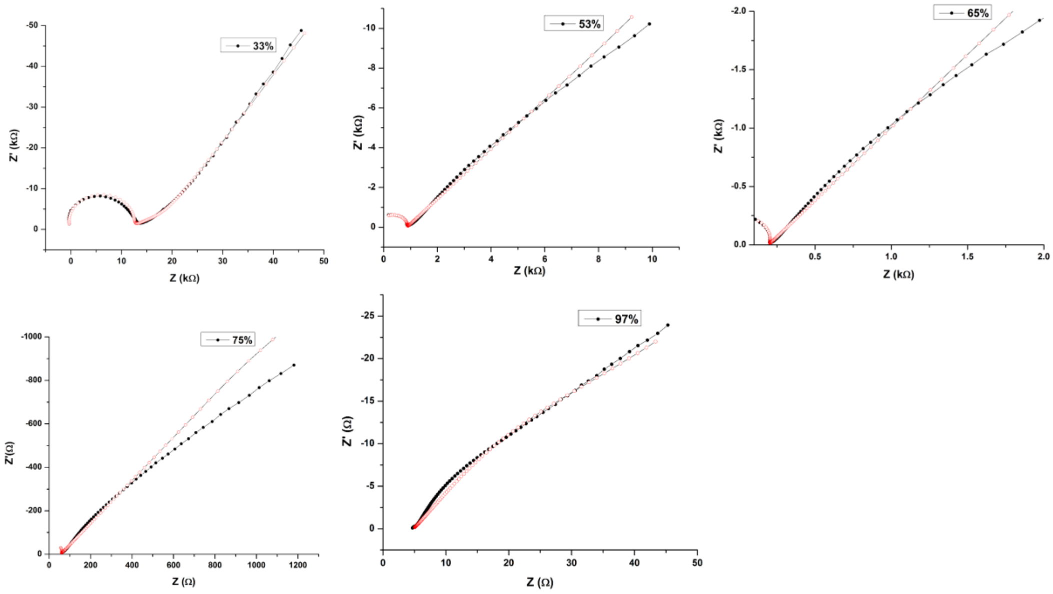

Figure S12. Nyquist plots for compound **2** at various relative humidity values at 20 °C; black dots are experimental data and red hollow dots are simulated data.

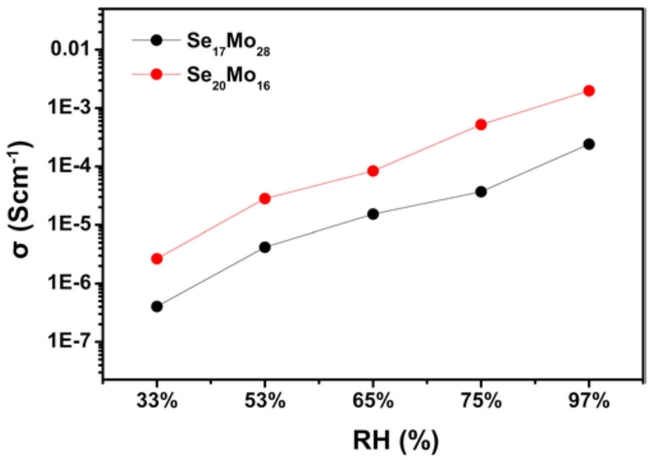

Figure S13. Comparison of the proton conductivity as a function of the relative humidity (RH) at 20°C for **1** (red) and **2** (black).
